# Cerebral Microemboli After TAVR: A Potential Clue to Solving the Enigma of Post-TAVR Stroke

**DOI:** 10.1016/j.jscai.2023.101216

**Published:** 2023-11-30

**Authors:** Rani K. Hasan, Hooman Bakhshi

**Affiliations:** Division of Cardiology, The Johns Hopkins University, Baltimore, Maryland

**Keywords:** cerebral microemboli, high-intensity transient signals, stroke, transcatheter aortic valve replacement, transcranial Doppler

Transcatheter aortic valve replacement (TAVR) is a revolutionary and disruptive technology that has profoundly affected the management of aortic valve disease since its inception in 2002. Despite the widespread success, growth, and iterative improvement of TAVR over the past 2 decades, stroke remains a nagging and potentially devastating complication. In 2019, the STS-ACC TVT Registry reported a 30-day post-TAVR stroke rate of 2.3%, down slightly from 2.75% in 2011.^1^ Several risk factors, such as older age, atrial fibrillation, severe aortic valve calcification, and atherosclerotic plaques in the aorta, for embolic stroke post-TAVR are described^2^; however, most of these risk factors are common in patients undergoing TAVR, and most of these patients will not experience stroke post-TAVR. Moreover, intraprocedural calcific and/or atheroembolic phenomena do not appear to be the only cause of post-TAVR stroke. Despite the successful deployment of a cerebral embolic protection device in 94.4% of patients in the PROTECTED TAVR trial, the incidence of periprocedural stroke was not significantly different among the cerebral embolic protection group and the control group.^3^ Hence, there are mechanisms of post-TAVR stroke, which remain undefined and may offer opportunities to further reduce this risk of this vexing and possibly catastrophic complication.

In this issue of *JSCAI*, Sharma et al^4^ present an observational pilot study in which they investigated the prevalence of neurovascular microemboli by high-intensity transient signals (HITS) measured using transcranial Doppler (TCD). In this single-center study, 51 patients underwent TCD pre-TAVR, intra-TAVR, and post-TAVR. HITS were not present in any of the patients pre-TAVR. Unsurprisingly, all patients demonstrated HITS during TAVR, which is consistent with previous studies. However, 56.8% of the subjects in this study demonstrated HITS 24 hours after TAVR; one of these patients had a stroke 48 hours post-TAVR. Furthermore, multivariable regression analysis did not demonstrate any association between clinical variables that are well known to predict intraprocedural microemboli, such as predilation, valve type, and valve-in-valve procedures, and post-TAVR HITS. Only absolute change in left atrial volume index was inversely associated with late HITS post-TAVR.

The study authors conjecture that the mechanism of late post-TAVR HITS that could underlie the risk of stroke may be subclinical aortic injury during TAVR with resultant in situ thrombus formation and late microembolization. This is a tantalizing hypothesis that warrants further investigation. If confirmed as a putative mechanism of post-TAVR stroke, this could lead to novel approaches to prevent these events. To be clear, this single-center pilot study is hypothesis-generating, and the study was not powered to detect an association between HITS and neurological events. HITS measured by TCD were used as a surrogate for microembolization in previous studies^5^^,^^6^; however, this technology cannot differentiate the size and composition of microemboli through the analysis of HITS. As the authors correctly note as a limitation of the study, cerebral imaging was not conducted in this study to determine whether HITS were associated with evidence of ischemic brain damage. Diffusion-weighted magnetic resonance imaging was shown in previous studies to detect new brain injury in ∼80% of patients after TAVR.^7^ In most cases, these patients remain asymptomatic in the short term, and it remains unclear whether these findings will have later clinical implications such as neurocognitive decline.^8^ Future research should include an adequate sample size to evaluate the association between post-TAVR HITS, clinical events, and imaging to evaluate the significance of microembolization regarding brain injury.

TAVR volumes will only continue to grow with the aging of the population and likely expansion in indications over time, underscoring the need to better understand and prevent the risk for post-TAVR stroke. Studies such as the present investigation reported by Sharma et al are important in illuminating potential new mechanisms for these presently unpredictable and unpreventable events. Such investigations will hopefully help to unravel the enigma of post-TAVR stroke and lead to effective strategies to prevent adverse neurologic events that can negate the benefits of successful TAVR.
